# A rapid realist review of patient engagement in patient-oriented research and health care system impacts: part one

**DOI:** 10.1186/s40900-021-00299-6

**Published:** 2021-10-10

**Authors:** Elaine Zibrowski, Tracey Carr, Shelagh McDonald, Heather Thiessen, Ray van Dusen, Donna Goodridge, Charlene Haver, Darcy Marciniuk, Christine Stobart, Tanya Verrall, Gary Groot

**Affiliations:** 1 Department of Community Health and Epidemiology, University of Saskatchewan, 107 Wiggins Road, Saskatoon, SK S7N 5E5 Canada; 2 Patient partner, Saskatoon, Saskatchewan Canada; 3 Patient partner, Regina, Saskatchewan Canada; 4 Saskatchewan Centre for Patient-Oriented Research, Health Sciences Building, 107 Wiggins Road, Saskatoon, SK S7N 5E5 Canada; 5 University of Saskatchewan, College of Medicine, 107 Wiggins Road, Saskatoon, SK S7N 5E5 Canada; 6 Saskatchewan Health Quality Council, Atrium Building, Innovation Place, 241 – 111 Research Drive, Saskatoon, SK S7N 3R2 Canada

## Abstract

**Background:**

Patient-oriented research affords individuals with opportunities to genuinely contribute to health care research as members of research teams. While checklists and frameworks can support academic researchers’ awareness of patient engagement methods, less guidance appears available to support their understanding of how to develop and maintain collaborative relationships with their patient partners. This knowledge is essential as patient partners report that the social atmospheres of research teams significantly impacts the quality of their experiences. This study sought to develop theory regarding how academic researchers support and sustain patient engagement in patient-oriented research.

**Methods:**

A six-step, rapid realist review was conducted: (1) research question development, (2) preliminary theory development, (3) search strategy development; (4) study selection and appraisal, (4) data extraction, analysis and synthesis (5) identification of relevant formal theories, and (6) theory refinement with stakeholders. Findings were additionally distilled by collective competence theory.

**Results:**

A program theory was developed from 62 international studies which illuminated mechanisms supporting academic researchers to engage patient partners, contexts supporting these mechanisms, and resources that enabled mechanism activation. Interaction between seven contexts (patient-oriented research belief, prior interaction with a healthcare system, prior interaction with a particular academic researcher, educational background of patient partner, prior experience with patient-oriented research, study type, and time lived in a rural-urban setting) and seven mechanisms (deciding to become involved in patient-oriented research, recognizing valuable experiential knowledge, cultural competence, reducing power differentials, respectful team environment, supporting patient partners to feel valued, and readiness to research) resulted in an intermediate outcome (sense of trust). Trust then acted as an eighth mechanism which triggered the final-level outcome (empowered patient-centred lens).

**Conclusions:**

Our theory posits that if patient partners trust they are a member of a supportive team working alongside academic researchers who authentically want to incorporate their input, then they are empowered to draw upon their experiential knowledge of health care systems and contribute as researchers in patient-oriented research. Our theory extends conceptual thinking regarding the importance of trust on patient-oriented research teams, how patient partners’ trust is shaped by team interactions, and the role that academic researchers have within those interactions.

**Supplementary Information:**

The online version contains supplementary material available at 10.1186/s40900-021-00299-6.

## Plain English Summary

Patient-oriented research gives patients, families, and caregivers opportunities to become members of health care research teams. Although academic researchers may be aware of what patient engagement is, they may not understand how to develop effective relationships with their patient partners. Academic researchers need this guidance because earlier research has shown that patient partners want to be supported to feel like they are important members of research teams. This support empowers them to feel confident to share their lived experiences and make suggestions and decisions about a research study. If patient partners believe their experiences and knowledge were not used or valued by academic researchers, then they may feel that their involvement was tokenistic. Tokenistic experiences discourage patient partners from participating in another research study.

We conducted a rapid realist review of 62 international studies to explore what works (and does not work) in patient-oriented research. This methodology supported us to examine existing research and better understand what contexts, how and why patient-oriented research led to outcomes on a health care system. The goal of this type of research study is to develop and refine a program theory that identifies how actions and activities lead to outcomes.

Our program theory emphasizes that patient partners need to trust the academic researchers they are working with. Several categories of actions (academic researcher’s behavior) helped researchers to gain the trust of their patient partners. Academic researchers were more (or less) likely to act in these ways depending on several contextual factors. Once patient partners trusted academic researchers on the team, they were empowered to draw upon their lived knowledge of health care systems and actively contribute as researchers. These findings are part of our complete theory about patient-oriented research impacts. They highlight why it is important to gain patient partners’ trust and how a complex set of actions are required by academic researchers to gain that trust.

**Supplementary Information:**

The online version contains supplementary material available at 10.1186/s40900-021-00299-6.

## Background

Patient-oriented research represents a paradigm shift in health care research [1–5]. Patient-oriented research aligns with moral, ethical and democratic principles advocating that the public has a fundamental right to be involved in research processes given that they are the ultimate end-user of healthcare research [6–9]. Rather than solely being sources of data for research studies, patient-oriented research provides patients, families, caregivers and other lay individuals with opportunities to join and genuinely contribute to health research teams (the public is collectively referred to as patient partners [2–4]).

In Canada and the United States, the central tenet of patient-oriented research is patient engagement [1, 4, 10]. While multiple interpretations of engagement exist [3, 4, 11], patient engagement in patient-oriented research intends to be a close equivalent of the United Kingdom’s concept of patient and public involvement [4]. These international definitions embrace patients as legitimate collaborators who take on active, meaningful roles alongside academic researchers, health care providers, and policy-makers in health research (professionals are collectively referred to as academic researchers [2, 10, 12]).

By bringing together individuals from diverse backgrounds who possess varying experiential and professional expertise, patient engagement adds a unique element to interdisciplinary teams engaged with health research practice. While checklists, frameworks and other tools are available to support academic researchers’ awareness of methods of engagement [13], less guidance is available to support them to understand how to develop and maintain effective collaborative relationships with lay researchers [14, 15]. Academic researchers need to develop new, and perhaps, unique skills to harness the benefit of involving patient partners on the research team because patient partners report that research teams’ social atmospheres significantly impact the quality of their experiences with patient-oriented research [16, 17]. Patient partners desire a team atmosphere to empower them to draw on and share their lived experiences with academic researchers [17]. Conversely, if patient partners perceive that their lived experiential knowledge is undervalued or under-utilized by academic researchers, they can believe their involvement in a study is tokenistic [18–20]. Tokenistic experiences discourage individuals from taking on future patient partner opportunities [16].

Limited efforts have been made to develop theory regarding the social processes that foster effective collaboration within patient-oriented research teams. Existing studies identified nurturing interpersonal relationships and feedback from researchers as mechanisms that supported teams to complete their work [21, 22]. Academic researchers developed relationships over time with their patient partners with effective communication and information sharing, utilizing both formal and informal group processes, being respectful and establishing trust [21, 22]. Feedback from academic researchers regarding their insight supported patient partners to feel they were valuable team members. These feelings were strengthened when patient partners saw how their contributions were incorporated into their team’s study [22].

While existing research has begun to illuminate the importance of social interaction within patient-oriented research teams, the lack of explicit theory regarding how patient engagement works, why, to what extent, and in what contexts continues to limit our ongoing capacity to implement successful patient-oriented research initiatives. The present study is part of a larger realist review which explored how, why, to what extent, and in what contexts does patient-oriented research produce impacts on individual collaborators, research processes, communities, and health care systems [23]. As our team worked on the data extraction, synthesis and analysis phases of our larger review, we recognized that our emerging program theory involved hypotheses regarding both the social processes that optimize collaboration on patient-oriented teams plus the impacts of patient partners’ involvement in patient-oriented research studies. In order to illuminate, and understand our full program theory, we felt it was best to present it within complementary articles. The present study aims to develop a program theory regarding how academic researchers support and sustain patient engagement in patient-oriented research. The guiding research questions were: 1) What mechanisms support academic researchers to engage patient partners on patient-oriented research teams; 2) What contexts support, or potentially hinder, these mechanisms; and 3) What resources support academic researchers involved in patient-oriented research to enable these mechanisms?

## Review

### Methodology

#### Study design – realist review methodology

Originally developed by Pawson and Tilley, a realist review is a type of narrative review that is grounded in the philosophical tradition of realism [24–29]. Realist reviews focus on interpretation, critique, and deepening of understanding of complex systems that occur across varied contexts and may be associated with varied, and potentially inconsistent, outcomes [29–32]. Realist reviews are not intended to provide generalized, prescriptive advice, but rather, seek to develop transferable program theory regarding when an intervention or program is more or less likely to be successful [26, 33].

A program theory is comprised of statements that link context, mechanism and outcome (commonly referred to as CMO [24, 28, 34]). Context refers to physical and social conditions that an intervention or program has been undertaken. Mechanisms are manifested as a shift in reasoning and/or response to the resources embedded in an intervention or program [24, 26, 27, 35–37] Outcomes are triggered by mechanisms. and an outcome can be explicit or hidden. Outcomes can involve intended or unintended consequences, and can be proximal, intermediate, or distal [35, 36]. Mechanisms can be explicit or hidden.

Realist reviews can follow a traditional or rapid approach. While both types involve the same research activities, a rapid realist review is intended to be responsive to limited time and resources for knowledge development around nascent issues [33]. Rapid realist reviews typically involve local stakeholders, including members of the lay public, during the review process. These stakeholders contribute local knowledge which, in turn, can potentially increase the transferability of the review’s findings [21, 33]. We selected the rapid review approach due to its emphasis on developing theory that can be valuable to policy-makers regarding an emerging issue, its recognition that members of the public should be considered as legitimate stakeholders, and that our study relied on a funding timeline of one year. Our review process was informed by the RAMESES guidelines and publication standards for a realist synthesis [38].

### Stages of a realist review

#### Stakeholder team and research question development

In January 2019, a 12-member stakeholder team met to discuss, and prioritize, potential patient-oriented research topics to explore with a realist review [23]. Team members brought expertise in health services research, lived illness experience, patient care, patient-oriented research, policy-making, qualitative inquiry, and realist research. Written field notes captured group dialogue during the meeting. After an additional consultation with an academic realist research group, the guiding research question for the review was refined, and approved, by the stakeholder team.

#### Preliminary program theory development and search strategy

A preliminary program theory was mapped into themes based on stakeholders’ dialogue from the January 2019 team meeting [Additional file 1: Appendix A]. Eight hypotheses were brought forth from themes related to patient engagement in patient-oriented research:

1. Moral and ethical ideals ground patient-oriented research [H1].
2. Patient-oriented research can reduce differences in power that exist between the public and their health care system [H2].
3. Members of the public are empowered to participate in patient-oriented research in response to a negative experience(s) with their health care system [H3].
4. Researchers are responsible for authentically engaging patient partners on a patient-oriented research team [H4].
5. Researchers and patient partners shape the social environment of a patient-oriented team [H5].
6. Differences in power can exist between patient-oriented team members [H6].
7. Patient partners can sense when a judgment is made by a researcher(s) regarding whether they are perceived as an authentic member of the research team. [H7].
8. Patient partners will draw on their lived experiences if their empowerment is sustained during a research study [H8].

In May 2019, stakeholder team members were asked to recommend information sources, both published and from the grey literature, to support an initial search of patient-oriented literature in CINAHL, Ovid MEDLINE, and Scopus. The preliminary theory plus full-text and references of recommended articles were used to identify a set of words, concepts, phrases, and author-derived keywords. Stakeholders were provided with iterations of search terms plus several abstracts from articles identified by a given pilot search. Search terms were combined using Boolean and proximity operators, and database subject headings, such as MeSH, were utilized [Additional file 1: Appendix B]. The public websites of international patient-oriented research initiatives were also reviewed. Covidence software© was used to de-duplicate, collate, and screen bibliographic records.

#### Study selection and appraisal

An iterative process was used to screen and appraise information sources, and to extract and synthesize data. Abstracts were screened by their title and abstract by two raters. Full-text versions of those sources were sought if both raters perceived the source met inclusion criteria [23]. The full-text of eligible sources were then appraised with Pawson’s criteria of rigour and relevance under a ‘fitness to purpose lens’ [25–28]. Rigour relates to the perceived methodological quality of a study, while relevance refers to the extent that a research study can potentially contribute information towards the development of theory for the realist review [25–28]. A source was appraised to be a good fit to the review if it appeared to be credible, trustworthy, and contained descriptive information that could potentially support the development of theory.

#### Data extraction, analysis, and synthesis

Each source was read line-by-line and information regarding the study’s design, how the patient-oriented research team unfolded their study, and main study outcome(s) were recorded in an Excel© spreadsheet. Descriptions of activities undertaken by individual team members and interactions that occurred between patient partners and academic researchers were also extracted.

Explanatory accounts were then developed from extracted data. These are causal statements that identify an enabling or a constraining factor(s) that were present in a study, the impact of those factors on one or more mechanisms, and the outcome(s) that were produced ([28, 35, 43, 44], Additional file 1: Appendix C).

The set of explanatory accounts were then iteratively analyzed in order to look for demi-regularities [35, 36, 44, 45]. Emerging findings were discussed by team members. Demi-regularities were then synthesized into thematic groups, and a context-mechanism-outcome configuration (CMO) was developed for each thematic group [35, 36]. The CMO configurations were then used to develop, and refine, an initial program theory.

The middle-range theory of collective competence was selected as an additional, substantive lens to distill the review’s findings. This theory posits that teamwork or ‘collective’ competence is distinct from individual competence [39–42]. Collective competence is context-dependent, and members of a given team learn to work together in an evolving, interdependent manner [41]. Developing collective competence requires social interaction, cooperation, shared experiences, and communication [39].

#### Theory refinement

The final stage of our rapid realist review involved the presentation and testing of the initial program theory with stakeholder team [25–28]. Three stakeholder meetings were held during the months of April and May 2020. During these meetings, stakeholders were asked to review and provide feedback on the initial program theory. The first meeting was prioritized for the voices of patient partners (session involved one stakeholder who facilitated dialogue with the three patient partners while another stakeholder recorded field notes; next two meetings were open to the entire team). Stakeholder feedback from each meeting was used to iteratively refine the initial theory. After the third stakeholder meeting, stakeholders concluded that additional re-examination of data was unlikely to result in any other major modifications to the CMO or refined program theory. At this point, the stakeholder team sensed theoretical saturation [25, 26].

## Results

### Document characteristics

After screening and quality appraisal of 2633 information sources, a final sample of 72 articles describin**g** 62 studies informed the review [several studies had multiple articles [2, 46–115, 119]; Additional file 1: Appendices D, E]. Twenty-two studies were from the United States (35.5%), 20 were from the United Kingdom (32.3%), 14 from Canada (22.6%), three from Australia (4.8%), and three involved international collaborations (4.8%). The studies were a mix of descriptive (52 studies, 83.9%) and interventional (10 studies, 16.1%) with diverse outcomes [Additional file 1: Appendices D, E].

### Analysis and synthesis

A grand total of 287 explanatory accounts were developed from the 62 studies. From these, 26 context-mechanism-outcome (CMO) configurations were identified and synthesized into an initial program theory. After review, feedback, and refinement by stakeholders, the revised program theory consisted of seven contexts and eight mechanisms [Additional file 1: Appendices F, G]. Five mechanisms supported the hypotheses put forth by the preliminary program theory.

Mechanisms were predominantly associated with sets of contexts. Interactions between contexts and the mechanisms “deciding to become involved in patient-oriented research [M1],” “recognizing valuable experiential knowledge [M2],” “cultural competence [M3],” “reducing power differentials [M4],” “respectful team environment [M5],” “supporting patient partners to feel valued [M6],” and “readiness to research [M7]” resulted in the intermediate outcome of a “sense of trust [_i_O].” Trust then acted as a mechanism [M8] which triggered the final outcome, “empowered patient-centred lens [_F_O]”. The following sections describe the set of eight mechanisms and their respective triggering contexts. Embedded resources that enabled each mechanism were identified. The relationships between mechanisms and the outcomes are presented below. Individual hypotheses that were supported from the preliminary program theory are identified.

### Deciding to become involved in patient-oriented research [M1]

Twenty-five articles revealed individuals are motivated to make a decision to become members of patient-oriented teams for various reasons [2, 46–48, 59, 65, 67, 68, 73, 74, 79, 81–83, 92, 93, 95, 97, 100, 107–111, 119]. Individually-centred motivations (potentially self-benefitting), and consistent with the first hypothesis [H1] of the preliminary program theory, altruistically-centred (potentially benefitting others) were identified for both patient partners and academic researchers. There was partial support of the third hypothesis [H3] in the preliminary program theory as both positive and negatively perceived health care experiences could motivate an individual to become a patient partner.

For patient partners, individually-centred motivations were the desire to learn new skills [68, 73, 110, 111] and to support the individual to reframe their self-identity away from being a sick person [73, 110, 111]. While honoraria did not appear to motivate individuals to participate in patient-oriented research, patient partner stakeholders of the review team further clarified that an unintended consequence of offering honoraria is that academic researchers may mistakenly perceive members of the public are predominantly drawn to patient-oriented research solely because of these funds.

Altruistically-centred motivations for patient partners included the desire to show gratitude and to ‘pay back’ their health care system for the good care they had received [107, 110, 111], desire to improve the living conditions of one’s community [74, 81, 92, 100, 107], and to improve the care of future patients [2, 47, 48, 59, 65, 73, 79, 82, 83, 93, 95, 97, 107, 110, 111]. Moreover, individuals can decide to become a patient partner after being a participant in a research study [46–48, 59, 108, 109, 119].

For academic researchers, individually-centred motivations were new opportunities for funding and for career advancement [73]. Altruistically-centred motivations included the potential for new knowledge creation, better understanding of patients’ perspectives, and improved patient care [73, 114].

Four articles identified disincentives for participating in patient-oriented research. Individuals who anticipated the work associated with being a patient partner or academic researcher would compete for time required in other aspects of their life, such as time required for family responsibilities, clinical duties, teaching, were less likely to participate in patient-oriented research [46, 67, 73, 107].

### Recognizing valuable, experiential knowledge [M2]

Active, meaningful engagement of a patient partner(s) within a research study assumes that an academic researcher(s) has made two decisions: the patient partner(s) possesses experiential knowledge that is relevant to the study, and it is anticipated that this knowledge can be utilized by the team in a manner that will improve that research study.

Nineteen articles revealed contexts that appeared to trigger these decisions for academic researchers [46–48, 53, 55, 57, 59, 70, 81, 84, 92, 93, 96, 100, 102, 108, 109, 112, 113]. These contexts included that the patient partner was a highly-regarded member of a group or community [47, 48, 53, 81, 84, 93, 100, 102, 112], the patient partner had lived within a rural or urban community for many years [47, 48, 59, 84, 92, 96, 113], the patient partner was a member of a recognized patient group or healthcare-related charity with a history of conducting research [55, 57, 70], the patient partner was a former study participant of the academic researcher [46–48, 59, 108, 109, 119].

### Developing cultural competency [M3]

Fourteen articles indicated the importance of cultural competency [46–48, 53, 66, 69, 73, 74, 79, 81, 87, 93, 95, 106]. Academic researchers who collaborate with vulnerable individuals and communities, but know little about the people, culture, and setting of their research, can face difficult interactions with groups and community members [53, 73, 74, 81, 87, 93]. For example, individuals who have endured discrimination and historical mistreatment can experience fear, anger, and/or be suspicious of researchers’ intentions in reaction to their presence within their community [73, 74, 87, 93]. Resources that supported academic researchers’ development of cultural understanding included their completion of cultural competency training [74, 87, 106], and their participation in a guided tour of the community early on during a study’s planning. During the tour, academic researchers listened to first-hand accounts from residents and community leaders about how their history and environment had negatively affected them [87]. Resources that supported patient partners to feel more comfortable included having academic researchers’ formally acknowledge to community leaders at the onset of study planning that historical mistreatment underlies community’s mistrust of their presence [87], having balanced representation of patient partners and academic researchers on the research team [74, 79, 87], rotating team meetings between community-based and academic-based locations [69] and providing informal opportunities for patient partners and academic researchers to try to get to know each such as sharing a meal prior to the start of a team meeting or taking time after a meeting ends to socialize with one another [46–48, 66, 74, 87, 95].

### Reducing power differentials [M4]

Twenty-three articles focussed on supporting groups and communities facing social inequities [46–48, 53, 65, 66, 68, 69, 72–74, 79, 81, 84, 87, 89, 93, 95, 97, 100, 102, 112, 114] Citizens of these groups and communities have endured historical discrimination and mistreatment, lived in impoverished communities and under-serviced areas, and/or are living with a stigmatized medical condition [47, 48, 53, 65, 68, 73, 81, 84, 87, 93, 97, 100, 102, 112, 114].

Consistent with the second and sixth hypothesis [H2, H6] of the preliminary program, a resource that afforded some power back to vulnerable groups and communities was the establishment of an advisory council which represented their collective interests during the study. Highly-regarded individuals within these groups and communities such as Indigenous tribal leaders, tribal elders, traditional healers, clergy, parents, and a rural mayor held membership on advisory councils [47, 48, 53, 81, 84, 93, 100, 102, 112]. Advisory committees made decisions throughout the course of a study granting of permission for outsiders to enter their community, providing insight to academic researchers regarding their community’s structure, and ensuring that activities introduced as part of the study were culturally-appropriate for their members. Several advisory councils also prepared a formal memorandum, or agreement, of understanding [47, 48, 72, 81, 100, 112]. This resource supported collective clarification of the roles, responsibilities, and deliverables that were required of the advisory council, patient partners, academic researchers and any other parties that were to be involved in the research study. A team may refer back to its memorandum of agreement if any questions or concerns arise [72].

Beyond the establishment of advisory councils, other resources were used to reduce perceived differentials in power between team members. These included having equal numbers of patient partners and academic researchers on the research team [74, 79, 87], rotating team meetings between community-based and academic-based locations [69] and requiring that academic researchers attend each team meeting [73]. Two sets of field notes, one recorded by patient partners and one recorded by academic researchers were included as official records of team meetings [89]. Informal activities such as sharing a meal before a team meeting afforded opportunities for team members to try to get to know each other [46–48, 66, 74, 87, 95].

### Respectful team environment [M5]

Consistent with the fourth hypothesis [H4] of the preliminary theory, ten articles indicated academic researchers used resources to cultivate, and maintain, team environments that were perceived by patient partners as being respectful and safe to participate in [2, 46, 63, 70, 73, 80, 97, 108, 109, 111]. Examples included scheduling research team meetings at times that were convenient for patient partners to attend [46, 73, 80], formally delegating protected time during team meetings where a patient partner(s) could speak to whatever topic they saw fit [108, 109], and recognizing that a patient partner’s medical condition can require them, in the moment, to have additional time to express their thoughts [80, 97]. Academic researchers also made efforts to explain medical jargon and avoid using acronyms as much as possible when interacting with their patient partners [46, 70]. Feelings of vulnerability can occur for patient partners who have endured discrimination and historical mistreatment, are living with a stigmatized medical condition, and/or are experiencing deteriorated health [2, 63, 110, 111]. During these moments, it was important patient partners were provided with emotional support from team members.

### Supporting patient partners to feel valued [M6]

Ten articles supported the hypothesis that patient partners are vigilant of cues from academic researchers that appeared to signal whether those academics valued them as authentic team members [66, 67, 74, 79, 80, 82–84, 108, 109]. Travelling to a patient partner’s home in order to meet [80], formally delegating protected time during research team meetings where a patient partner(s) was free to talk about topic(s) they perceived as being important [108, 109], and providing patient partners with individual honoraria and/or covering their travel and childcare costs [66, 67, 79, 84, 108, 109] were actions made by academic researchers that demonstrated to patient partners that their input was valued. A community-based organization could also be recognized with an honorarium [74].

The value of patient partners’ expertise was also memorialized on study-related brochures and other text-based information sources. These information sources identified patient partners as official members of a care team [82, 83], announced ongoing achievements made by members of a community during a study [81] and repeatedly emphasized that patient partner expertise enabled the study to thrive [97]. Patient partners were also formally identified as a co-investigator or co-author of the study [66, 108, 109].

### Readiness to research [M7]

The preliminary program theory did not take into account patient partners’ preparedness for patient-oriented research. Four articles indicated that when moments of tension were reported between academic researchers and patient partners, these appeared to be triggered by academic researchers’ perceptions regarding patient partners’ lack of methodological knowledge and/or inexperience with processes required for the study ([73, 85, 87, 119]; Additional file 1: Appendix C).

Twenty articles revealed that educational background and previous experience with patient-oriented research were contexts that appeared to signal, to academic researchers, that an individual was either ‘research ready’ or would likely require only minimal training to become ready to research [53, 55, 57, 60, 62, 63, 68–70, 73, 76, 82–85, 87, 90, 96, 97, 106]. Educational backgrounds signaled a patient partner was research ready if the individual had previously completed training in a reputable, patient-oriented research program [76], the individual was a member of a recognized patient group or healthcare-related charity with a history of conducting research [55, 57, 70]. Individuals with a medical degree or medical background were also perceived as being research ready [97]. Academic researchers provided training during the course of the study in order to prepare patient partners without research backgrounds [53, 60, 62, 63, 68, 69, 73, 82–84, 87, 90, 96, 106]. The review team’s patient partner stakeholders further advocated the advancement of patient partner expertise requires that individuals have ongoing opportunities for collaboration with academic researchers in patient-oriented research.

### Developing a sense of trust [_i_O_1_/M8] and empowering a patient-oriented lens [_F_O]

Fifteen studies revealed that the development of a sense of trust was important for maintaining cooperative relationships between patient partners and academic researchers [47, 48, 66, 67, 72, 74, 79, 84, 87, 95, 102, 106, 108, 109, 119]. Resources used by academic researchers to develop patients’ sense of trust included providing honoraria to patient partners [66, 67, 79, 84, 108, 109], seeking the consensus of all team members during decision-making [106], incorporating patient partner input into the study [108, 109], making visible efforts to try to get to know members of the community where a study was set [47, 48], and being willing to engage in open, frank discussion with community members about their intentions in the community [74].

While the preliminary program theory emphasized empowerment of patient partners [H3, H8], it did not overtly position a role for trust. The review team’s patient partner stakeholders recognized, and emphasized, this was an important omission because just as patients, families, and caregivers need to perceive a sense of trust during any interaction with their health care system, trust also lies at the centre of the patient partner-academic researcher relationship. These stakeholders cautioned that if patient partners do not develop a sense of trust with academic researchers, a patient-oriented research study will not achieve its full potential. This is because members of the public, including experienced patient partners, will not be empowered to feel confident to fully open up to researchers regarding their lived health journeys, how they perceive their personal health experiences can be of benefit to the research study, and make suggestions and decisions about a research study.

## Discussion

This rapid realist review outlined mechanisms which enabled academic researchers to develop trusting relationships with patient partners; recognizing valuable experiential knowledge, cultural competence, reducing power differentials, respectful team environment, supporting patient partners to feel valued, and readiness to research. These mechanisms interacted with contexts, and resources used by academic researchers to enable activation of mechanisms were identified. Our program theory posits that if patient partners trust they are part of a supportive research team working alongside academic researchers who authentically want to incorporate their input, then they are empowered to draw upon their experiential knowledge of health care systems and contribute as researchers in patient-oriented research.

Consistent with other studies, our review found that patients, families, and caregivers are empowered to become involved in patient-oriented research in order to better themselves and for the greater good of others [116–118]. Once they have joined patient-oriented research teams, their empowerment can be supported and sustained by academic researchers’ behavior. While other studies have identified the importance of developing trust between team members [21, 22], our rapid realist review extends conceptual thinking regarding the facets of trust, how trust is shaped for patient partners by both formal and informal team interactions, and the role that academic researchers have within those interactions.

While an emerging area of patient-oriented research involves identifying competencies from the perspective of individual stakeholders [5], the formal theory of collective competence reminds us that most work involves complex sets of activities performed by individuals and groups [39–41]. For this reason, a solely individualistic stance regarding competence is inadequate, and this theory incorporates an additional team-based lens.

Collective competence is context-dependent, and members of a given team learn to work together [41]. To be “collectively competent” teams’ enact three processes dependent on communication; making collective sense of events, developing a collective knowledge base, and maintaining a sense of interdependency [41]. While these processes do not fully explain how patient partners are empowered, they do provide support towards six mechanisms within our program theory.

A team needs to develop a collective understanding of the goal(s) of their joint activity [41]. Team members co-construct their understanding of what they wish to achieve by engaging in task talk [41, 110]. Dialogue can also reduce team members’ feelings of uncertainty, doubt and fear. Collective understanding underpins the establishment of advisory councils seen in the mechanism “reducing power differentials.” Advisory councils were a resource that afforded vulnerable communities to negotiate a shared understanding of the roles, responsibilities and deliverables of a research study with academic researchers. Meeting minutes and formal memorandum or agreements of understanding served as artifacts of co-constructed understanding.

Teams also need to develop a collective knowledge base that their members can draw on throughout their work [41]. Collective knowledge requires team members’ share their individual expertise with each other. By doing so, the teams’ level of consolidated knowledge exceeds knowledge brought by its individual members. Expertise of individual team members may introduce multiple lay and professionalized languages to the team. In order to avoid misunderstandings, discussion of language differences between team members is important [41, 110]. The mechanisms “recognizing valuable experiential knowledge,” “developing cultural competence,” “respectful team environment,” and “readiness to research” are supported by development of collective knowledge. Academic researchers sought patient partners from varied contextual backgrounds because they anticipated these individuals would contribute valuable experiential knowledge to their research team. Academic researchers also attempted to better understand the people, culture, and setting of their research study by undergoing didactic cultural competency training and listening to first-hand accounts from members of a vulnerable community. Academic researchers reserved time for patient partners to speak freely during team meetings. They also explained medical jargon to lay users and avoided using acronyms.

Academic researchers’ explicit acknowledgment to community members that they understood the community had endured historical mistreatment and understanding why a patient partner’s medical condition can require them to need extra time to express their thoughts were artifacts of collective knowledge. While academic researchers provided training to patient partners who did not have research backgrounds, the moments of tension that arose in some teams due to patient partners’ apparent lack of methodological expertise signalled, conversely, the team may have under-developed shared understanding in this area.

Most teams organize their work with a division of labour. Team members need to cooperate and share a sense of interdependency [40, 41]. Interdependency becomes fragmented when team members act without considering each other’s needs or the team’s purpose [40]. Resources that triggered the mechanisms “reducing power differentials,” “supporting patient partners to feel valued,” and “readiness to research” were supported by a sense of interdependency. Advisory councils monitored the involvement of academic researchers with members of vulnerable communities. Academic researchers enabled patient partners’ participation with a visit to their home, scheduling meetings that took into account their availability, and provided an individual honorarium and/or reimbursement of travel and childcare costs. Academic researchers also provided training to patient partners without research backgrounds. Memoranda of agreement, announcements that emphasized the importance of patient partner expertise, and formally identifying a patient partner as a co-investigator or co-author were artifacts of the interdependency between academic researchers and patient partners.

### Practical implications

The program theory advanced in this rapid realist review presents an opportunity for increasing understanding of the complexity involved in patient-oriented research. Our program theory emphasizes the importance of developing patient partners’ trust, how trust is shaped for patient partners as they collaborate with academic researchers, and its relationship to patient partners’ empowerment. If patient partners do not develop trust in the academic researcher members on the team, they will not feel confident to fully open up to academic researchers regarding their lived health journeys and how they perceive these experiences can be of benefit to the research study, and make suggestions toward and decisions about it.

We anticipate that our program theory will support researchers, clinicians, and policy-makers, including individuals working with national-level patient-oriented research initiatives, to reflect on their practices of engaging patient partners, and in combination with their own experiences and insight into their own contexts, to be able to refine their strategies for patient engagement. We also advocate that designers of training for patient-oriented research consider advancing their curriculum regarding the mechanisms that support academic researchers to optimize their collaborative relationships with lay researchers. This education may be especially important for researchers who have limited experience interacting with patients, families, and caregivers.

### Future research

We have developed a novel program theory which reveals academic researchers undertake a complex set of actions and responsibilities as they attempt to support and sustain the engagement of patient partners in patient-oriented research. We acknowledge the dissemination of our program theory is a first step to the application of our findings by other researchers. We anticipate future research will test the transferability of our program theory. Future research should explore, and illuminate, the development of academic researchers’ trust of patient partners.

### Strengths and limitations

Patient partners were full research partners and their engagement was informed by an engagement framework co-designed with patient partners by the provincial-level, patient-oriented research support centre [120].

Patient partners supported the development of our guiding research question, recommended literature to contribute to our review, and reviewed our set of search terms. During theory refinement, patient partners were asked to review, evaluate and reflect on our initial program theory in light of their personal, lived expertise. Stakeholder feedback, including feedback from patient partners, was used to iteratively refine the initial theory. Patient partners also contributed to the conceptualization and writing-review of the manuscript. Patient partners were offered honoraria, and their travel expenses were reimbursed. Honoraria and travel were funded consistent to provincial guidelines.

As with any other review article, our research cannot comprehensively consider the entire discourse for patient-oriented research. Our review was limited to English-disseminated information sources. Realist reviews are not intended to identify pathways to achieving all possible outcomes, nor can this methodology exhaustively explain all mechanisms that are capable of generating changes.

## Conclusions

Using rapid realist review methodology, we have conducted a realist review of the social processes utilized by academic researchers to support and sustain engagement of patient partners in patient-oriented research. We have disseminated a program theory, which identified mechanisms that can support academic researchers to foster an optimal team environment when collaborating with members of the lay public. Our findings allow us to better understand how interactions with academic researchers affect the empowerment of patient partners within patient-oriented research.

## Supplementary Information

**Additional file 1.** Appendices A-G.

## Data Availability

Data supporting the findings of this study are available from the lead author upon a reasonable request.

## Abbreviations

CMO: context-mechanism-outcome configuration
