# Supplementary material for: A rapid realist review of patient engagement in patient-oriented research and health care system impacts: part one
Source: Res Involv Engagem. 2021 Oct 10;7:72. doi: 10.1186/s40900-021-00299-6 (PMC8504114; doi:10.1186/s40900-021-00299-6)
Supplement: Supplementary file 1 — Additional file 1. Appendices A-G. [file 40900_2021_299_MOESM1_ESM.zip › 40900_2021_299_MOESM1_ESM/APPENDIX A.docx]

| Why does patient-oriented research (POR) exist? |
| --- |
| - Moral and ethical imperatives guide POR |
| - POR was created because “it’s the right thing to do” - Patients/families can be let down, ignored, misjudged by their health care |
| - “No research about me, without me.” - Patients are stakeholders of their own care |
| - Patients have a right to meaningfully contribute to research |
| - POR gives patients a chance to improve their health care |
| - Some individuals feel that POR is the only way they can potentially improve their health care system |
| Themes: POR is morally and ethically grounded by affording opportunities for patients, families, caregivers the opportunity to improve their health care system. Patients, families, caregivers are empowered by a negative experience(s) with a health care system to participate in POR. POR can afford some power back to members of the public to potentially improve their health care system [POR can reduce the power differential that exists between patients, family, caregivers, other members of the public and their health care system] |
| Hypotheses: POR is grounded by moral and ethical ideals. POR can reduce differences in power that exists between the public and their health care system. Members of the public are empowered to participate in POR in response to a negative experience(s) with their health care system. |
|  |
| What are some essential elements of POR? |
| - All POR involves a partnership between patients and researchers |
| - In order to partner with patients, researchers need to engage them in research |
| - Engagement means active, meaningful involvement. Not tokenistic involvement - POR is not merely being a ‘named’ as a team member - Patients want to be authentically involved in research |
| - Successful POR means that the partnership between patients and researchers was successful |
| Themes: Researchers engage patient partners [it is researchers’ responsibility to engage their patient partners]. True engagement of a patient partner requires that individual to be authentically involved in the research study or project [researcher responsibility]. |
| Hypothesis: Researchers are responsible for authentically engaging patient partners on POR team. |
|  |
| What can be an experience of being patient partner in POR? |
| - Being a patient partner can be an intimidating experience - It can be first time they interact with professionals who make their health care decisions. It's not your typical medical appointment |
| - Patient partners perceive early on if researchers don’t really see them as authentic members of the team |
| - Experienced patient partners can also feel intimidated depending upon the team |
| Themes: Power differentials can exist between researchers and patient partners who are members of a POR team. Each team shapes its own social environment [because even experienced patient partners can feel intimidated]. Patient partners are vigilant towards cues from researchers about whether they perceive a patient partner should be part of their team [patient can sense that a judgement has been made regarding the value they can bring] |
| Hypotheses: The social environment of a POR team is shaped by its members. Difference in power can exist between POR team members. Patient partners can sense when a judgment is made by a researcher(s) regarding whether they are perceived as an authentic member of the research team. |
|  |
| What can POR achieve? |
| - Engagement allows patients to provide insight about what matters to them |
| - Patient engagement improves research |
| - Improved research leads to impacts within a health care system |
| Themes: Empowered patient partners bring their lived insight to POR [they are empowered to seek involvement in POR]. POR is a pathway to positive impacts for a health care system. POR improves research- research improves health care [therefore, patient partner empowerment needs to be sustained] |
| Hypothesis: Patient partners will utilize their lived experiences if their empowerment is sustained during a research study. |
