# Supplementary material for: A rapid realist review of patient engagement in patient-oriented research and health care system impacts: part one
Source: Res Involv Engagem. 2021 Oct 10;7:72. doi: 10.1186/s40900-021-00299-6 (PMC8504114; doi:10.1186/s40900-021-00299-6)
Supplement: Supplementary file 1 — Additional file 1. Appendices A-G. [file 40900_2021_299_MOESM1_ESM.zip › 40900_2021_299_MOESM1_ESM/APPENDIX B.docx]

| **Search syntax for Cinahl** |
| --- |
| ( ( TITLE-ABS-KEY ( ( "health care"  OR  healthcare )  W/1  ( organization  OR  service*  OR  system ) ) )  AND  ( TITLE-ABS- AND  ( ( TITLE-ABS-KEY ( ( "community participation"  OR  "stakeholder participation" )  W/1  research ) )  OR  ( TITLE-ABS-KEY ( ( consumer*  OR  public*  OR  policy*  OR  participat*  OR  "service user*" )  W/1  research ) )  OR  ( TITLE-ABS-KEY ( "patient-oriented research"  OR  "patient research partner*"  OR  coresearcher*  OR  "co-researcher*" ) ) ) ) |
|  |
| **Search syntax for Ovid Medline** |
| 1. ("patient-oriented research" or "patient research partner*" or (coresearcher* or "co-researcher*") or ((Community Participation or Stakeholder Participation) and research) or ((public or policy* or participat*) adj1 research)).ti,ab,kw. |
| 2. ("Delivery of Health Care" and (organization or service* or system)).ti,ab,kw. |
| 3. (Health Services and (organization or system)).ti,ab,kw. |
| 4. 2 or 3 |
| 5. (impact or codesign or "Quality of Health Care").ti,ab,kw. |
| 6. 1 and 4 and 5 |
|  |
| **Search syntax for SCOPUS** |
| 1.  ( TITLE-ABS-KEY ( ( "health care” OR healthcare) W/1 ( organization  OR  service*  OR  system ) ) ) |
| 2.  ( TITLE-ABS-KEY ( impact  OR  "quality improvement" ) ) |
| 3. ( ( TITLE-ABS-KEY ( ( "community participation"  OR  "stakeholder participation" )  W/1  research ) )  OR  ( TITLE-ABS-KEY ( ( consumer*  OR  public*  OR  policy*  OR  participat*  OR  "service user*" )  W/1 research ) )  OR  ( TITLE-ABS-KEY ( "patient-oriented research"  OR  "patient research partner*"  OR  coresearcher*  OR  "co-researcher*" ) ) ) ) |
| 4. 1 AND 2 AND 3 |
