# Supplementary material for: A rapid realist review of patient engagement in patient-oriented research and health care system impacts: part one
Source: Res Involv Engagem. 2021 Oct 10;7:72. doi: 10.1186/s40900-021-00299-6 (PMC8504114; doi:10.1186/s40900-021-00299-6)
Supplement: Supplementary file 1 — Additional file 1. Appendices A-G. [file 40900_2021_299_MOESM1_ESM.zip › 40900_2021_299_MOESM1_ESM/APPENDIX C.pptx]

## Slide 1
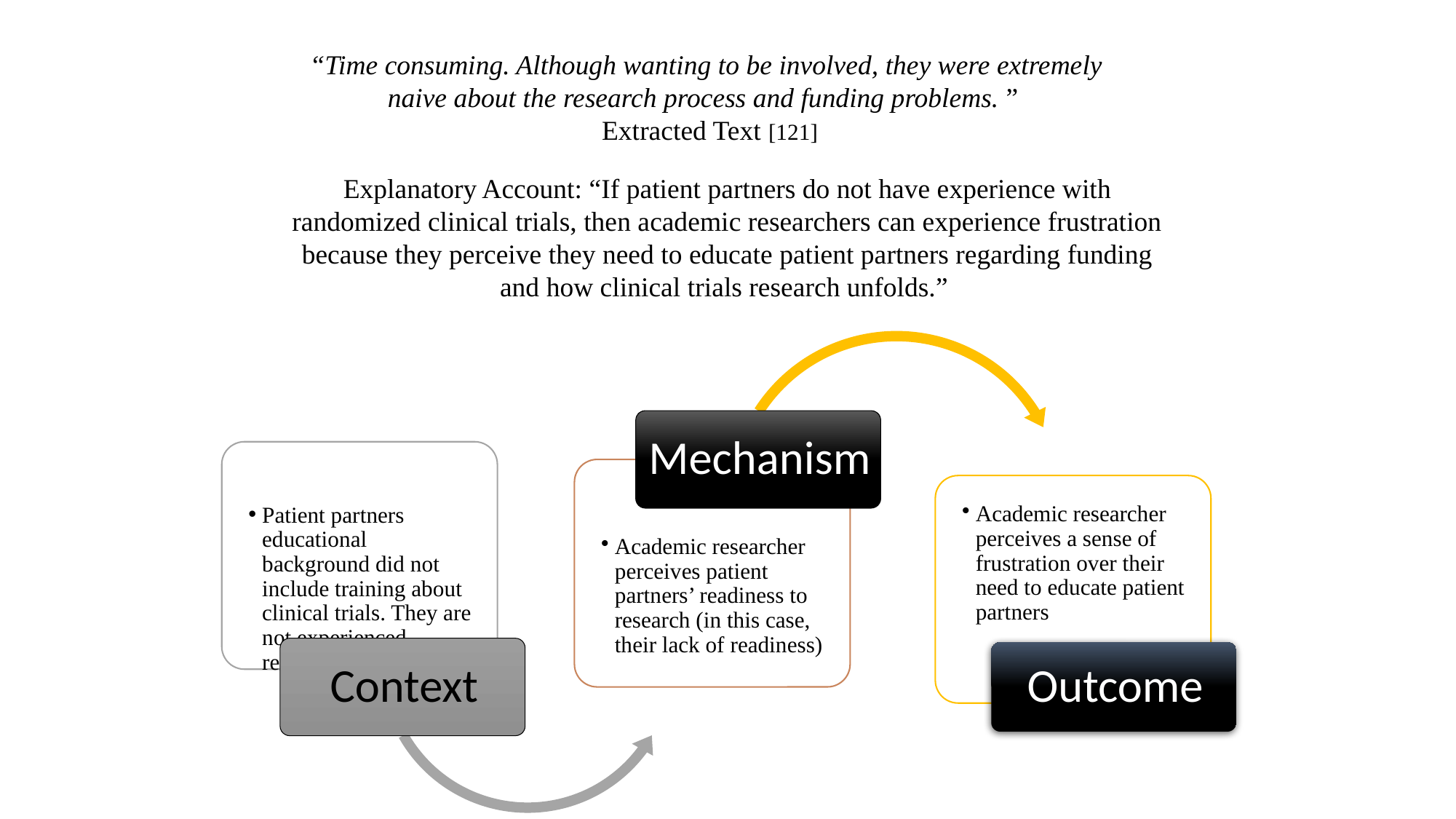

“Time consuming. Although wanting to be involved, they were extremely naive about the research process and funding problems. ”
 Extracted Text [121]
Explanatory Account: “If patient partners do not have experience with randomized clinical trials, then academic researchers can experience frustration because they perceive they need to educate patient partners regarding funding
and how clinical trials research unfolds.”
