# Supplementary material for: A rapid realist review of patient engagement in patient-oriented research and health care system impacts: part one
Source: Res Involv Engagem. 2021 Oct 10;7:72. doi: 10.1186/s40900-021-00299-6 (PMC8504114; doi:10.1186/s40900-021-00299-6)
Supplement: Supplementary file 1 — Additional file 1. Appendices A-G. [file 40900_2021_299_MOESM1_ESM.zip › 40900_2021_299_MOESM1_ESM/Appendix D.docx]

| Lead Author, Publication Year(s) | Country | Study Type | Participants | Main Study Outcome(s) |
| --- | --- | --- | --- | --- |
| Absolom 2015 | United Kingdom | Qualitative with narrative description | 18-member research advisory group | Development of a research advisory group aimed at including patient and public involvement for patient-reported outcomes research |
| Bacsu 2017  2019 | Canada | Community-based using interviews, observation, concept mapping | 43 seniors living rural Saskatchewan | Increased understanding of cognitive health concerns and supported needs identified by healthy seniors living in rural communities. were prioritized by interviewees |
| Boote 2002  Boote 2016 | United Kingdom | Cluster Randomized Clinical Trial | 142 general practices across England and Wales whose patients included children living with asthma | There was no evidence of effect in terms of unscheduled contacts in September. Among children aged 5–16 years, the odds ratio (OR) was 1.09 [95% confidence interval (CI) 0.96 to 1.25] against the intervention. The intervention did increase the proportion of children collecting a prescription in August (OR 1.43, 95% CI 1.24 to 1.64) as well as scheduled contacts in the same month (OR 1.13, 95% CI 0.84 to 1.52). The intervention did not reduce unscheduled care in September, although it succeeded in increasing the proportion of children collecting prescriptions in August as well as having scheduled contacts in the same month |
| Breault 2018 | Canada | Priority-setting | 445 individuals with living with depression | Increased understanding of depression research questions prioritized by patients living with depression |
| Caress 2010 | United Kingdom | Mixed-methods involving a questionnaire, individual and focus group interviews | 354 survey participants (253 patients and 101 family members); 20 individual interviewees (13 patients and seven family members), and 30 patient participants in focus groups | Increased understanding of health literacy of individuals diagnosed with chest problems and their family members regarding their risk of swine flu infection |
| Cashman 2008_1 Chino 2006 | United States | Participatory action research | Six to eight health staff plus one school board member who represented tribal leadership and ad-hoc group of six to eight representatives from schools, police services, the Ramah Navajo Tribal Chapter, Tribal community elders, University faculty and staff | Increased understanding of the Ramah Navajo capacity to perform ten essential health services |
| Cashman 2008_2 Parker 1998 | United States | Participatory action research | 700 community residents | Identification of the health services prioritized by community members as requiring improvements to community access and quality improvement |
| Cashman 2008_3 | United States | Participatory action research | Seven focus groups with non-English-speaking, less-acculturated Latino men | Increased understanding of the sociocultural determinants of sexual risk for non-English-speaking, less-acculturated Latino men |
| Cashman 2008_4 | United States | Participatory action research | One team with four scientists and 20 community members | Increased understanding of the numbers and locations of previously unidentified polluting facilities, such as dry cleaners, nail salons, and off-set printers, whose proximity to residential buildings was potentially dangerous. Elevated concentrations of percholoroethylene, toluene, and xylene outside of homes and elevated concentrations readings of organic compounds (VOC) inside apartments were detected. Elevated ambient levels of VOC were also found on streets where trucks were illegally transporting through the community |
| Cawston 2007 | United Kingdom | Participatory action research | 444 individuals living in an underserviced, peripheral housing estate in rural Scotland | Increased understanding of individuals' perspectives regarding how health services could be improved for their community |
| Clemens 2019 | Canada | Qualitative with individual interviews and focus groups | 12 patients living with diabetes | Increased understanding of risks and challenges faced by high-risk diabetes patients with co-morbidities |
| Cook 2013 | United Kingdom | Participatory action research | 43 service users, 24 staff members,  23 family members/carers, 8 voluntary sector representatives | Increased understanding of the perspectives of service users and family members regarding their engagement with service provision |
| Corner 2007  Wright 2006 | United Kingdom | Priority-setting | 105 patients living with varying cancers | Increased understanding of the research priorities important to cancer patients |
| Cunningham 2015 | United Kingdom | Randomized Clinical Trial | 615 infants with physician-diagnosed bronchiolitis | The primary outcome was time to cough resolution. There was equivalence at the prespecified variance of ± 2 days [time to cough resolution: standard care group, 15 days; modified care group, 15 days; median difference 1 day (benefit modified), 95% confidence interval (CI) –1 to 2 days] |
| Davidson 2010 | Canada | Community-based participatory research | Working group with youth, adults, provincial leaders from mental health and addictions services, child and family development, and a ministry of health | Formation of a provincial-level family research council for child and youth mental health |
| de Wit 2015 | 12 European countries | Validation of a patient reported outcomes instrument | 703 psoriatic arthritis patients involved in ranking and prioritizing item domains, cognitive debriefing interview, and a validation study | Validation of a Psoriatic Arthritis Impact of Disease Score |
| DeCamp 2015 | USA | Multimodal, mixed methods evaluation | 22 Latino mothers whose children were cared for at a paediatric clinic | Formation of a family advisory board to support improving the health services for a paediatric clinic serving predominantly Latino families with limited English proficiency |
| Durham 2019 | Australia | Participatory action research | 31 young Pasifika individuals aged 16–24 years old | Increased understanding regarding the multiplicity of place-based factors that interact in complex ways to shape health inequities for young Pasifika peoples |
| Edgren 2005 | USA | Community-based research with interviews | 105 community members of a low-income community in Detroit | Community members were important to the recruitment, data collection, retention of families in a study of childhood asthma triggers. Seventy-eight percent of the families recruited by community members were retained during the first year of the study |
| Edwards 2011  Wyatt 2011 | United Kingdom | Randomized Clinical Trial | 142 children aged 5–12 years living with cerebral palsy | Compared with children in the control group, children in the osteopathy group demonstrated no statistically significant differences in GMFM-66 (mean difference 4.9, 95% CI −4.4 to 14.1), CHQ Physical Summary Score (mean difference 2.2, 95% CI −3.5 to 8.0) or CHQ Psychological Summary Score (mean difference 3.4 , 95% CI −0.8 to 7.7). There were no significant differences between groups with respect to pain; sleep (either ‘time asleep’ or ‘time to sleep’); or main carers quality of life. Compared with children in the control group, carers of children receiving cranial osteopathy were nearly twice as likely to report that their child’s global health had ‘improved’ at 6 months rather than ‘decreased’ or ‘remained the same’ (38% vs 18%; odds ratio 2.8, 95% CI 1.1 to 6.9) |
| Eisinger 2001 | United States | Participatory action research | One urban research centre staffed by epidemiologists, anthropologists, a sociologist, an economist, a community organizer, and supporting staff | Development of an urban research centre to conduct community-driven investigative research and evaluation |
| Flicker 2008 | Canada | Participatory action research | 34 youth living with HIV/AIDS | Development of four youth-friendly, self-published, non-commercial magazines for community distribution. Team members also collaborated on the development of three community newsletter articles |
| Flores 2006 | USA | Qualitative with dramatic performance | Very large coalition including Latino community-based organizations, health clinics, social service agencies, faith-based groups, and employee-based organizations, migrant health clinics, and scientific partners | Development of a bilingual play, "Cancer Monologues,” intended to increase community awareness of risks for cancers |
| Gill 2016 | Canada | Qualitative with focus groups and individual interviews | 32 former intensive care patients or family members of surviving or deceased patients who received intensive care | Increased understanding of patient and family experiences during admission, daily care while in the intensive care, and after discharge from hospital |
| Gillard 2010 | United Kingdom | Qualitative with secondary analyses of historical interview data | Historical interviews of psychiatric patients detained under the 1983 Mental Health Act | Increased understanding of how service user researchers differ from university researchers in their coding and interpretation of mental health patient interviews |
| Greenwood 2019 | USA | Case study | Publicly-available Twitter posts from 150 members of a diabetes online community | Development of an intercultural diabetes online community plus a community advisory board |
| Hanes 2019 | Canada | Qualitative with focus group interviews | 16 individuals aged 17 to 29 living with cerebral palsy | Increased understanding of the lived experience of young people with cerebral palsy regarding their physical, mental, and emotional health |
| Hanley 2001 | United Kingdom | Survey | 62 contacts at trial coordinating centres and 60 investigators of randomized controlled trials involving consumers | Increased understanding of investigators’ perceptions of the prevalence and impact of involving consumers in the coordination of randomized clinical trials |
| Jinks 2009 | United Kingdom | Case study | One team with lay users, university researchers, and community service and volunteer organization workers | Development of a community knee pain forum to research prevention of knee pain prevention |
| Kearns 2020 | United Kingdom | Questionnaire development | Six individuals living with aphasia | Development of a questionnaire to better understand experiences of individuals living with aphasia when engaging with information communication technology-delivered rehabilitation |
| Kelley 2018 | Canada | Comparative case study | Four First Nations communities in Manitoba and Ontario | Development of community advocate groups to discuss how palliative care at home can be delivered in a culturally-appropriate manner |
| Khan 2017  2018 | USA, Canada | Prospective before and after study | Patients, resident physicians, nurses, and medical students from seven paediatric inpatient hospitals | Development of a co-designed, standardized healthcare provider-family communication on ward rounds. The family-centered rounds reduced harmful medical errors decreased by 38% after its implementation |
| Kleinman 2011 | United States | Participatory action research | Very large coalition involving public agencies, local health care, state health departments, social services agencies, grassroots organizations, local business leaders, community residents and leaders | Increased understanding of how a structured, built environment (built environment) led a city borough to become a diabetogenic environment |
| Krewulak 2019 | Canada | Single-centre, pilot tool validation study | 17 intensive care patient and family dyads | Proof of concept that family caregivers can complete a questionnaire of delirium detection in an intensive care setting. |
| Lechelt 2018 | Canada | Priority-setting | 161 patients living with head and neck cancers | Increased understanding of the most important treatment uncertainties to a patients and family members living with head and neck cancers and the clinicians who manage and support their treatment |
| Lewis 2015 | USA | Qualitative | Four teams comprised of academic and non-academic stakeholders | Development of a mini-research school to increase cultural competency in academic researchers and research skill awareness in non-academic stakeholders |
| McCormick 2019 | Canada | Cross-sectional, online survey | 151 individuals recruited from patient partners' affiliated organizations | Increased understanding of individuals perspectives regarding the use of data obtained from publicly-funded healthcare for research purposes |
| Mendel 2011 | USA | Program evaluation | Two underserved areas in Los Angeles County | Increased awareness regarding an upcoming randomized clinical trial on depression care improvement |
| Middleton 2011 | United Kingdom | Qualitative with individual interviews | 33 individuals who had recently used the services of crisis resolution home treatment | Increased understanding of the experiences of mental health patients who recently received the services of a crisis home treatment |
| Mishra 2018 | USA | Qualitative with interviews | 60 clinicians and eight managers from two, established accountable care organizations | Increased understanding of education needs for clinicians and managers regarding patient activation |
| Munoz 2013 | United Kingdom | Participatory action research | One team of community members, health care providers and commissioners | Increased understanding of perspectives of community members who co-designed health and care services for their rural community |
| Nelson 2018 | USA | Clinical trial | 98 Zuni Indians living with chronic kidney disease | Patients who received the home program were 4.8 times more likely to have activation score of at least 3 (they were "taking action") regarding their health. Body Mass Index declined by 1.1 kg/m2 (P=0.01), hemoglobin A1c declined by 0.7% (P=0.01), high-sensitivity C-reactive protein declined by 3.3-fold (P,0.001), and the Short-Form 12 Health Survey mental score increased by five points (P=0.002) in the intervention group relative to usual care |
| Oliver 2001 | United Kingdom | Pilot study | 10 healthcare consumers | Increased understanding of how healthcare consumers prioritized research topics and reviewed draft research proposals |
| Patel 2016 | United States | Case study | 37 individuals from varied professions | Increased understanding of how a citizen scientist model can be an effective strategy to meaningfully engage underrepresented populations in clinical and translational research |
| Peacock 2011 | USA | Program evaluation | Six outreach workers in two Chicago IL communities of high-risk for perinatal and infant morbidity and mortality | Increased understanding about the complex lives of underserved women at risk for poor pregnancy outcomes and the challenges and rewards of the outreach worker role |
| Pelletier 2015 | Canada | Participatory action research | 146 patient participants with serious mental illness and chronic, comorbid condition(s) | Development of a questionnaire plus 33 short videos intended to support patients with serious mental illness to prepare for an appointment with their family physician |
| Potestio 2015 | Canada | Qualitative content analysis | 38 members of the public | Increased understanding of patient and family perspectives regarding how to potentially improve communication between intensive care patients and their families with health care providers |
| Rai 2018 | Canada | Qualitative with focus group interviews | 27 individuals with rheumatoid arthritis | Increased understanding of methods preferred by patients for receiving information about rheumatoid arthritis and support for medication adherence |
| Ritte 2016 | Australia | Participatory action research | 323 individuals representing 107 organizations (30% of organizations were Indigenous); 38.7% of participants were Indigenous individuals | Development of a social movement aimed at supporting aboriginal mothers during the first 1000 days of their child’s life |
| Saunders 2007 | Australia | Development of research appraisal instrument | 14 individuals who were members of a cancer advocacy group, a member of a financial donor list for cancer research, or a lay member of the public | Development of a research appraisal instrument for service users to provide an appraisal towards recommendations of research funding |
| Smith 2002 | USA | Community-based research with focus groups | 101 individuals living in rural communities in West Virginia | Development of collaborative relationships with community gate-keepers that could enable researchers to gain entry to communities otherwise inaccessible |
| Staddon 2013 (INVOLVE) | United Kingdom | Qualitative with individual interviews | 23 females with alcohol issues | Increased understanding of how women with alcohol issues perceive their support could be improved |
| Staniszewska 2012  Brett 2011 | United Kingdom | Systematic mapping review | Systematic mapping review of 72 interventions for communicating with a parent(s) of a preterm infant | Increased understanding of effective interventions to support parents of preterm infants |
| Stirman 2010 | USA | Qualitative with interviews, document review | Interviews with clinicians, administrators, staff and research personnel, review of meeting minutes and grant materials | Development of a partnership between a community mental health organization and a team of researchers for depression research. Physical environment of the treatment area and waiting room of a depression clinic was improved |
| Suarez-Balcazar 2006 | USA | Qualitative with narrative description | Multi-disciplinary team involving four academic institutions and three community-based organizations | Formation of an interdisciplinary partnership aimed at increasing access to healthier foods and better nutrition |
| Sweeney 2005 | United Kingdom | Qualitative with interviews | 28 individuals from nine NHS Trusts | Increased understanding of how best to involve patients in planning and improving the quality of care that they receive |
| Tapp 2017a  2017b | United States | Prospective cohort study | 200 children and 106 adults living with asthma | Proportion of pediatric patients with one or more exacerbations was significantly lower in the shared decision making (SDM) intervention group compared to controls during 12 months after exposure to the intervention (33% vs. 47%, p = 0.023). For adults, there was not a strong association between use of the SDM intervention and outcomes improvement |
| Thompson 2009  Thompson 2014 (INVOLVE) | United Kingdom | Ethnography | 14 individuals who had participated in patient and public involvement research | Increased understanding of motivations that draw service users to participate in patient and public involvement research |
| Wahbe 2007 | Canada and Ecuador | Participatory research | Two Indigenous communities (one in Canada, one in Ecuador) | Development of a partnership between two Indigenous communities which afforded the identification of relevant health concerns pertinent to each group |
| Wells 2013 | United States | Cluster-randomized matched  design | 1,246 individuals living with depression | The collaborative model improved individuals' mental health related quality of life and mental wellness, physical activity, and lowered an indicator for being homeless or having multiple risk factors for future homelessness |
| Woolf 2016 | United States | Case study | 72,000 patients people facing decisions about breast, colorectal, and prostate cancer screening | Development of an online module to support patients' decision-making regarding their readiness for cancer screening |
| Wyatt 2008 | United Kingdom | Case study | Consumers, service providers and academics who had collaborated on service improvement research | Increased understanding of consumer collaborators experiences |
