# Supplementary material for: A rapid realist review of patient engagement in patient-oriented research and health care system impacts: part one
Source: Res Involv Engagem. 2021 Oct 10;7:72. doi: 10.1186/s40900-021-00299-6 (PMC8504114; doi:10.1186/s40900-021-00299-6)
Supplement: Supplementary file 1 — Additional file 1. Appendices A-G. [file 40900_2021_299_MOESM1_ESM.zip › 40900_2021_299_MOESM1_ESM/Appendix E.pptx]

## Slide 1
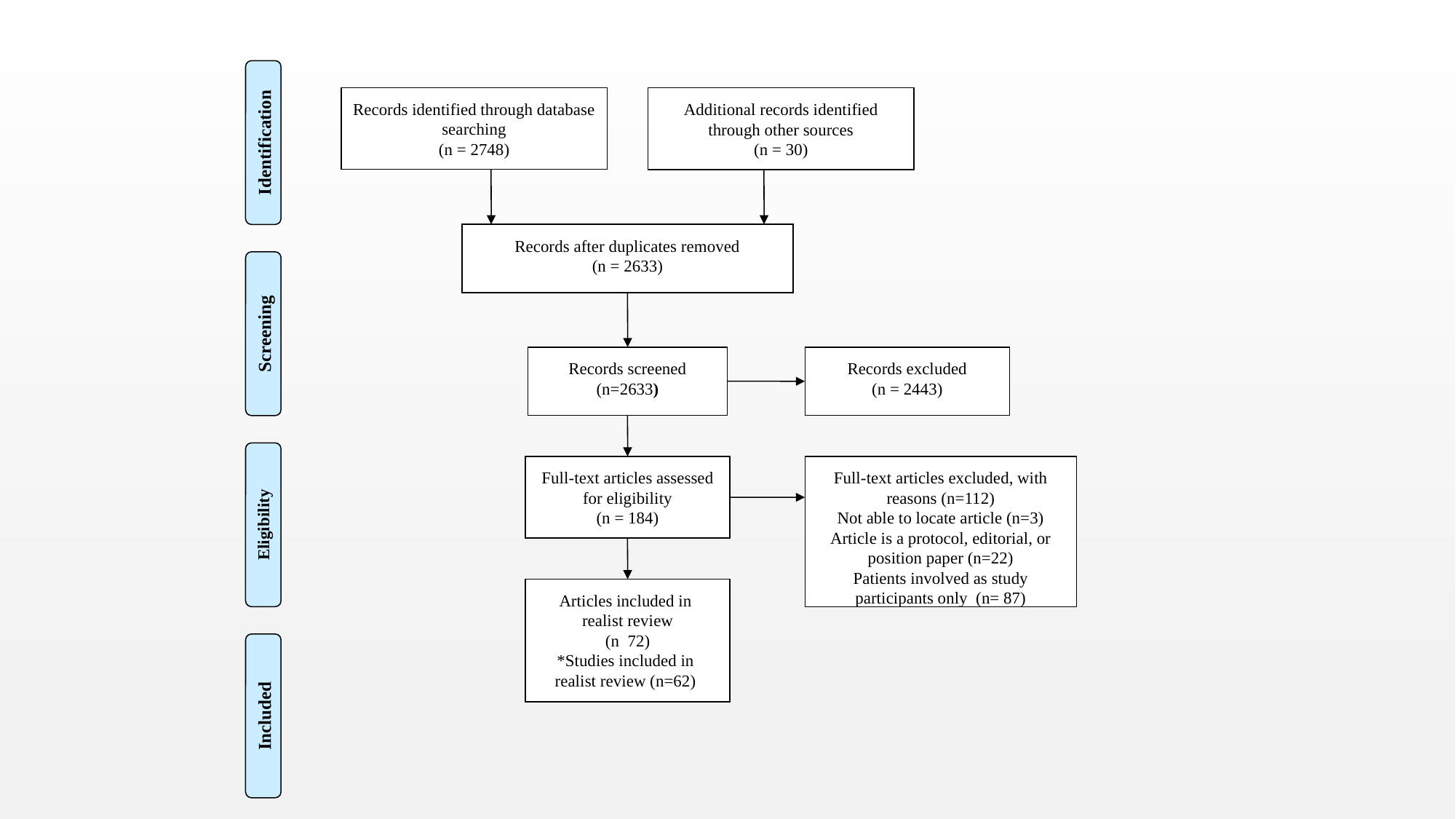

Records identified through database searching
(n = 2748)
Additional records identified through other sources
(n = 30)
Identification
Records after duplicates removed
(n = 2633)
Screening
Records screened
(n=2633)
Records excluded
(n = 2443)
Full-text articles assessed for eligibility
(n = 184)
Full-text articles excluded, with reasons (n=112)
Not able to locate article (n=3)
Article is a protocol, editorial, or position paper (n=22)
Patients involved as study participants only (n= 87)
Eligibility
Articles included in
realist review
(n 72)
*Studies included in
realist review (n=62)
Included
