# Supplementary material for: A rapid realist review of patient engagement in patient-oriented research and health care system impacts: part one
Source: Res Involv Engagem. 2021 Oct 10;7:72. doi: 10.1186/s40900-021-00299-6 (PMC8504114; doi:10.1186/s40900-021-00299-6)
Supplement: Supplementary file 1 — Additional file 1. Appendices A-G. [file 40900_2021_299_MOESM1_ESM.zip › 40900_2021_299_MOESM1_ESM/APPENDIX F.docx]

| Context (C1) | | Patient-oriented research belief | | **Mechanism 1: Deciding to become involved in patient-oriented research [M1]** |
| --- | --- | --- | --- | --- |
| Context (C1) | | Patient-oriented research belief | | Desire to be involved in an activity that will support the individual to reframe their identity from being a sick person (C) + deciding to become involved in patient-oriented research (M) ➔ more likely to become a patient partner in patient-oriented research (O) |
| Context (C1) | | Patient-oriented research belief | | Desire to be involved in an activity that will be of benefit to an individual’s community (C) + deciding to become involved in patient-oriented research (M) ➔ more likely to become a patient partner in patient-oriented research (O) |
| Context (C1) | | Patient-oriented research belief | | Desire to be involved in an opportunity that will of benefit to an academic researcher (C) + deciding to become involved in patient-oriented research (M) ➔ more likely to become an academic researcher in patient-oriented research (O) |
| Context (C1) | | Patient-oriented research belief | | Individual perceives that time required to participate in patient-oriented research will compete/take away from time required for other responsibilities + deciding to become involved in patient-oriented research (M) ➔ less likely to become a patient partner in patient-oriented research study conducted by that particular academic researcher (O) |
| Context (C2) | | Prior interaction with a health care system | | Previous health care experience that was perceived as being positive (C) + deciding to become involved in patient-oriented research (M) ➔ more likely to become a patient partner in patient-oriented research (O) |
| Context (C2) | | Prior interaction with a health care system | | Previous health care experience was perceived as being negative (C) + deciding to become involved in patient-oriented research (M) ➔ more likely to become a patient partner in patient-oriented research (O) |
| Context (C3) | | Prior interaction with a particular academic researcher | | Previous involvement as a participant in research study carried out by a particular academic researcher (C) + deciding to become involved in patient-oriented research (M) ➔ more likely to become a patient partner in patient-oriented research study conducted by that particular academic researcher (O) |
|  | |  | | **Mechanism 2: Recognizing valuable experiential knowledge [M2]** |
| Context (C3) | | Prior interaction with a particular academic researcher | | Previous involvement as patient partner on a patient-oriented research study with a particular academic researcher (C) + value of experiential knowledge (M)➔academic researcher has increased perception that patient partner(s) will bring valuable experiential knowledge (O) |
| Context (C3) | | Prior interaction with a particular academic researcher | | Previous involvement as a participant in a research study carried out by a particular academic researcher (C) + value of experiential knowledge (M)➔academic researcher has increased perception that patient partner(s) will bring valuable experiential knowledge (O) |
| Context (C5) | | Previous experience with patient-oriented research | | Patient partner is a member of a recognized patient research group or charity that conducts research (C) + value of experiential knowledge (M)➔academic researcher has increased perception that patient partner(s) will bring valuable experiential knowledge (O) |
| Context (C6) | | Study type | | Study is set within a community or group of marginalized individuals, individuals who have experienced discrimination, individuals with stigmatized health conditions (C) + value of experiential knowledge (M) ➔academic researcher has increased perception that patient partner(s)from that community or group will bring valuable experiential knowledge (O) |
| Context (C7) | | Time lived in rural-urban setting | | Patient partner has lived in rural or urban community for many years (C) + value of experiential knowledge (M) ➔academic researcher has increased perception that patient partner(s) will bring valuable experiential knowledge (O) |
|  | |  | | **Mechanism 3: Cultural competency [M3]** |
| Context (C6) | | Study type | | Study is set within a community or group of marginalized individuals, individuals who have experienced discrimination, individuals with stigmatized health conditions (C) + cultural competency (M) ➔patient partner(s) and academic researcher perceive that academic researcher has increased understanding of beliefs, customs, values, experiences that exist within a culture (O) |
|  | |  | | **Mechanism 4: Reducing power differentials [M4]** |
| Context (C4) | | Educational background of patient partner | | Patient partner does not have post-secondary education (C) + reduction in power differentials (M) ➔ patient partner is more likely to perceive balanced power amongst patient partners and academic researchers (O) |
| Context (C5) | | Prior experience with patient-oriented research | | Individual does not have experience as a patient partner with a patient-oriented research group or study (C) + reduction in power differentials (M)➔ patient partner is more likely to perceive balanced power amongst patient partners and academic researchers |
| Context (C6) | | Study type | Study is set within a community or group of marginalized individuals, individuals who have experienced discrimination, individuals with stigmatized health conditions (C) + reduction in power differentials (M) ➔ patient partner more likely to perceive balanced power amongst patient partners and academic researchers (O) | |
|  | |  | **Mechanism 5: Cultivating a respectful, supportive environment [M5]** | |
| Context (C4) | | Educational background of patient partner | | Patient partner does not have post-secondary education (C) + cultivating a respectful, collaborative environment (M) ➔ patient partner has increased perception that research team environment is supportive and safe (O) |
| Context (C6) | | Study type | | Study is set within a community or group of marginalized individuals, individuals who have experienced discrimination, individuals with stigmatized health conditions (C) + cultivating a respectful, collaborative environment (M) ➔ patient partner has increased perception that research team environment is supportive and safe (O) |
|  | |  | | **Mechanism 6: Supporting patient partner to feel valued [M6]** |
| Context (C5) | | Previous experience with patient-oriented research | | Individual does not have experience as a patient partner with a patient-oriented research group or study (C) + supporting patient partner to feel valued (M)➔ patient partner has increased perception of being a valued member of the research team (O) |
| Context (C5) | | Previous experience with patient-oriented research | | Individual has previous experience as a patient partner with a patient-oriented research group or study (C) + supporting patient partner to feel valued (M)➔ patient partner has increased perception of being a valued member of the research team (O) |
| Context (C6) | | Study type | | Study is set within a community or group of marginalized individuals, individuals who have experienced discrimination, individuals with stigmatized health conditions (C) + supporting patient partner to feel valued (M)➔ patient partner has increased perception of being a valued member of the research team (O) |
|  | |  | | **Mechanism 7: Readiness to research [M7]** |
| Context (C4) | | Educational background of patient partner | | Patient partner has post-secondary education (C) + readiness to research (M) ➔academic researcher has increased perception that patient partner is ready to be involved in research work needed to complete the study (O) |
| Context (C5) | | Previous experience with patient-oriented research | | Individual has previous experience as a patient partner with a patient-oriented research group or study (C)+ readiness to research (M) ➔academic researcher has have increased (perception that patient partner is ready to be involved in research work needed to complete the study (O) |
| Context (C5) | | Previous experience with patient-oriented research | | Patient partner is a member of a recognized patient research group or charity that conducts research (C) readiness to research (M) ➔academic researcher has increased self-perception that they are ready to be involved in research work needed to complete the study (O) |
|  | |  | | **Mechanism 8: Sense of trust [M8]/Intermediate-level outcome[_i_O_1_]** |
| Contexts (C1…C7)  Mechanisms (M1…M7) | |  | | (C1…C7) + (M1…M7)➔ Patient partner perceives an increased sense of trust in their working relationship with an academic researcher(s) ➔ (M_8sense of trust_) ➔ (O_intermediate_sense of trust)_ |
|  | |  | | **Final-level outcome: Empowered patient-centred lens** |
|  | |  | | (C1…C7) + (M1…M8) ➔ (O_Final_empower lens_) partner perceives is empowered to draw upon their experiential knowledge and contribute to a patient-oriented research study |
