# Supplementary material for: A rapid realist review of patient engagement in patient-oriented research and health care system impacts: part one
Source: Res Involv Engagem. 2021 Oct 10;7:72. doi: 10.1186/s40900-021-00299-6 (PMC8504114; doi:10.1186/s40900-021-00299-6)
Supplement: Supplementary file 1 — Additional file 1. Appendices A-G. [file 40900_2021_299_MOESM1_ESM.zip › 40900_2021_299_MOESM1_ESM/APPENDIX G.pptx]

## Slide 1
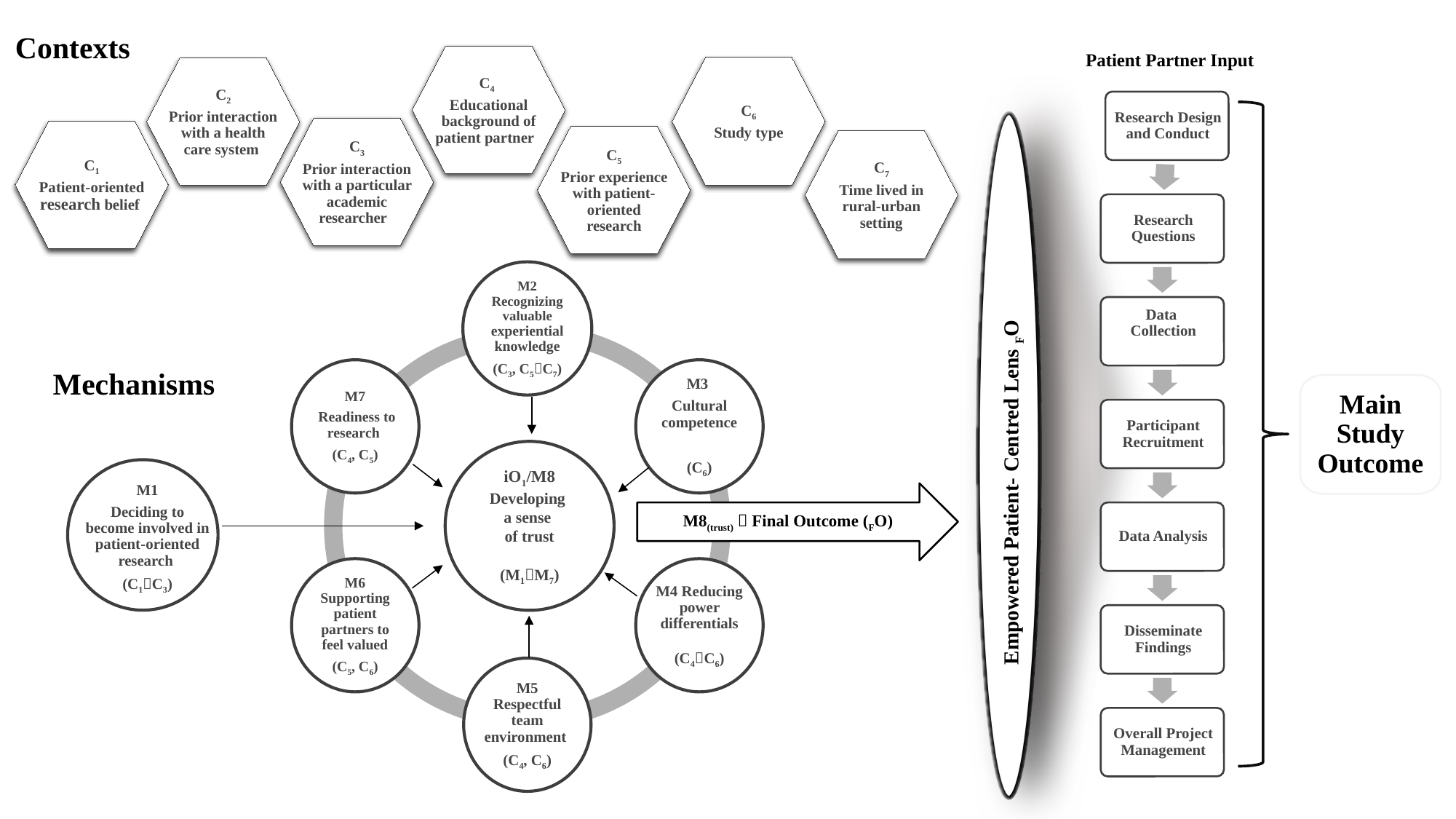

C7
Time lived in rural-urban setting
C6
Study type
C5
Prior experience with patient-oriented research
C4
Educational background of patient partner
C3
Prior interaction with a particular academic researcher
C2
Prior interaction with a health care system
C1
Patient-oriented research belief
Contexts
Patient Partner Input
Mechanisms
Main Study Outcome
M1
Deciding to become involved in patient-oriented research
(C1C3)
Empowered Patient- Centred Lens FO
M8(trust)  Final Outcome (FO)
